# How Users Experience and Use an eHealth Intervention Based on Self-Regulation: Mixed-Methods Study

**DOI:** 10.2196/10412

**Published:** 2018-10-01

**Authors:** Louise Poppe, Celien Van der Mispel, Geert Crombez, Ilse De Bourdeaudhuij, Helene Schroé, Maïté Verloigne

**Affiliations:** 1 Research Group Physical Activity and Health Department of Movement and Sports Sciences Ghent University Ghent Belgium; 2 Ghent Health Psychology Lab Department of Experimental-Clinical and Health Psychology Ghent University Ghent Belgium

**Keywords:** eHealth, self-regulation, behavioral change theory, interview, usage data

## Abstract

**Background:**

eHealth interventions show stronger effects when informed by solid behavioral change theories; for example, self-regulation models supporting people in translating vague intentions to specific actions have shown to be effective in altering health behaviors. Although these theories inform developers about which behavioral change techniques should be included, they provide limited information about how these techniques can be engagingly implemented in Web-based interventions. Considering the high levels of attrition in eHealth, investigating users’ experience about the implementation of behavior change techniques might be a fruitful avenue.

**Objective:**

The objective of our study was to investigate how users experience the implementation of self-regulation techniques in a Web-based intervention targeting physical activity and sedentary behavior in the general population.

**Methods:**

In this study, 20 adults from the general population used the intervention for 5 weeks. Users’ website data were explored, and semistructured interviews with each of the users were performed. A directed content analysis was performed using NVivo Software.

**Results:**

The techniques “providing feedback on performance,” “action planning,” and “prompting review of behavioral goals” were appreciated by users. However, the implementation of “barrier identification/problem solving” appeared to frustrate users; this was also reflected by the users’ website data—many coping plans were of poor quality. Most users were well aware of the benefits of adopting a more active way of living and stated not to have learned novel information. However, they appreciated the provided information because it reminded them about the importance of having an active lifestyle. Furthermore, prompting users to self-monitor their behavioral change was not sufficiently stimulating to make users actually monitor their behavior.

**Conclusions:**

Iteratively involving potential end users offers guidance to optimally adapt the implementation of various behavior change techniques to the target population. We recommend creating short interventions with a straightforward layout that support users in creating and evaluating specific plans for action.

## Introduction

eHealth, or “the use of technology to improve health care” [1] is effective in changing health behaviors, such as increasing physical activity, altering dietary habits, and smoking cessation [2-4]. Furthermore, eHealth programs have the potential to reach large populations in a cost-effective way [5-7]. They may also enable a personalized and interactive approach, for example, by computer tailoring, without the practical considerations of face-to-face contacts [7-9].

There are strong indications that eHealth interventions should be informed by sound theories. Research has shown that applying a theoretical basis to eHealth interventions increases their effectiveness [10,11]; for example, self-regulation models [12] have identified several techniques that may help users to engage in behavioral change. Self-regulation is the process of goal selection, pursuit, and maintenance [13]; it focuses not only on eliciting an intention to change behavior but also on bridging the gap between intention and behavior [13-15]. Using the self-regulation perspective, individuals may learn how to initiate change effectively and how to maintain health behavior over changing conditions. “Action planning,” for example, comprises the detailed planning of what a person will do, whereas “barrier identification/problem solving” helps individuals to identify and solve difficult situations for performing the health behavior [16]. Furthermore, research has shown that self-regulation strategies are, indeed, effective in changing behavior [17-21].

Although behavioral change theories inform us about which behavioral change techniques should be included, they provide limited information about how these techniques can be implemented in an engaging way [10]; this might explain why Web-based and mobile interventions often suffer from high attrition rates (60%-80%) [22-24]. The use of behavioral change theories may be necessary but not sufficient to guarantee efficacious interventions. Equally important is the involvement of potential users during various stages of the development process. Such an approach has been advocated by many and is known as cocreation [25], person-based approach [26], or user-centered development [27].

Involving the target population has given researchers insight into what motivates users to start and adhere to a Web-based intervention; for example, Bardus et al. found that the expectation of receiving reminders regarding physical activity was an important reason to start with a Web-based physical activity intervention [28]. Time efficiency, a clear navigation structure, and professional design of the eHealth intervention have been shown to be important factors to make users stay in the program [29,30]. Finally, providing users with a sense of control motivates them to complete the eHealth program [31]. These findings act as a guide to further fine-tune eHealth interventions to the target population [26].

This study aims to investigate how users experience self-regulation techniques implemented in an eHealth intervention. For this purpose, we used the eHealth intervention “MyPlan 2.0,” which supports users to be more physically active or less sedentary in a step-by-step manner. This intervention is informed using self-regulation theory and considers users as their own expert in the behavioral change process. Through a semistructured interview and an examination of users’ website data, information was obtained about the appreciation of the website and intervention in general and the experience of users with various self-regulation techniques (ie, goal setting, providing information, providing feedback on performance, action planning, barrier identification/problem solving, prompting self-monitoring, planning social support, and reviewing behavioral goals). The findings derived from this study might help other eHealth developers on how (not) to implement self-regulation techniques in Web-based interventions.

## Methods

### Participants

In this study, 20 adults from the general population volunteered to participate; this number was based on previous qualitative research about eHealth by Yardley et al. [32]. Participants were recruited via acquaintances of the researchers and a database of the research group. The database contained the names of persons who expressed interest in participating in studies of the Ghent Health Psychology Research Group. The exclusion criteria were as follows: not having internet access, aged <18 years, diagnosed with a chronic disease, and non-Dutch speaking. To maintain an equal distribution over age, gender, and educational level, we preselected participants based on these characteristics. The study was conducted between November 2016 and May 2017. As soon as a participant was enrolled in the study, he or she could start the intervention. The first participant started in November 2016, and the last participant started in April 2017. The study was approved by the Committee of Medical Ethics of the Ghent University Hospital (Belgian registration number: B670201629995), and all participants provided a written informed consent.

### Intervention

“MyPlan 2.0” is a self-regulation-based intervention consisting of 5 weekly Web-based sessions. It aims to increase physical activity and decrease sedentary behavior in adults and is designed and created by our research group. “MyPlan 2.0” is based on a previous version named “MyPlan 1.0” [33], which was effective in changing health behaviors [21,33-35]. However, the quantitative research with “MyPlan 1.0” revealed high levels of nonusage attrition [36]. The qualitative research revealed that users felt frustrated about the length and complexity of the program [30]. Hence, the intervention was iteratively transformed according to this feedback. In particular, the intervention was shortened, the text was limited, information sheets were substituted by a quiz, and the layout was changed. Furthermore, rationales were provided for the implementation of different self-regulation techniques, specific instructions were given during action planning and barrier identification/problem solving, and general tips and tricks were provided. Moreover, success stories of other users were added.

**Figure 1:**
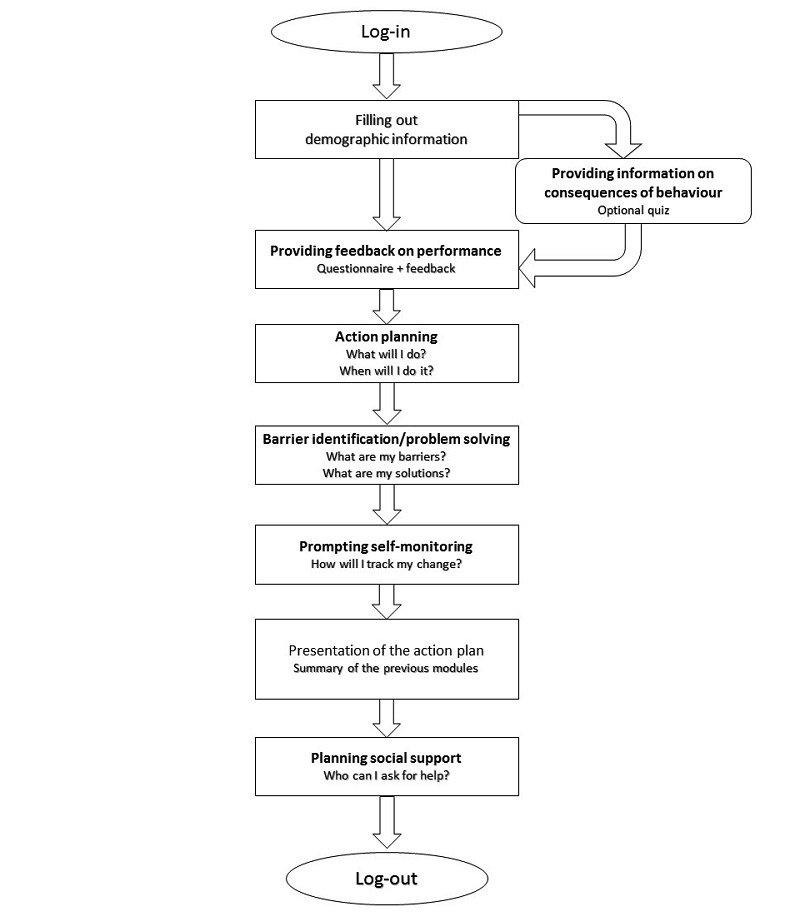
Flowchart of the first session.

In the first session, participants started by creating a profile and provided general information (eg, gender, age, and working status) to enable personalized messages during the intervention. In addition, they chose which behavior, physical activity or sedentary behavior, they wanted to change during the intervention (ie, “goal setting”). The website offers the option to take a quiz regarding the chosen health behavior (ie, “providing information on the consequences of the behavior”). Thereafter, participants completed a short questionnaire regarding the selected health behavior, that is, a shortened version of the International Physical Activity Questionnaire (IPAQ) [37] or a last 7-days sedentary behavior questionnaire [38] and received tailored feedback, that is, “providing feedback on performance.” After receiving feedback, participants were guided to the “action planning” technique. During this component, users specified their actions in terms of what, where, and how by answering open- and multiple-choice questions. Several tips were provided to make the action plan feasible (eg, “Choose for one goal instead of multiple goals, this increases the chance of goal attainment”). Next, “barrier identification/problem solving” was introduced by asking users which barriers they could perceive and which solutions were possible to overcome these barriers. In addition, examples of barriers and related solutions were provided, which could be selected by users. Next, “prompting self-monitoring of behavior” was introduced. Users chose from a list how they would monitor their behavior (eg, via their calendar, in a notebook, and so on). During the action planning, barrier identification/problem solving, and self-monitoring component, success stories from fictitious users were shown; these were incorporated to elicit motivation further and provide inspiration. At the end of the first session, “planning social support” was introduced; users read about how to elicit social support, how to talk about behavioral change to significant others, and how to find opportunities to engage in behavioral change together with other people. Figure 1 depicts the flowchart of the first session. Multimedia Appendix 1 shows the exact implementation of the techniques through screenshots.

**Figure 2:**
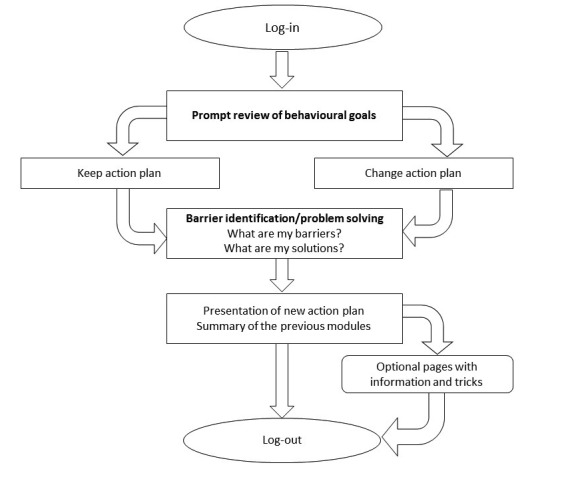
Flowchart of the follow-up sessions.

After 1 week, users received an email to return to the eHealth program to revise their plan. According to the technique “Prompt review of behavioral goals,” they were asked how well the behavioral change was going and whether they wanted to adapt or maintain their plan. If they wanted to adapt their plan, action planning was again completed. In all cases, users were prompted for barrier identification and problem solving. To motivate users to think about more personally relevant barriers and solutions, users now answered an open-ended question instead of selecting an option from a predefined list. A summary of their answers was shown in the action plan, and users were prompted to self-monitor their behavior. In addition, users could again read the information about social support and receive extra tips and tricks, and this illustrated the use of different self-regulation techniques, such as “prompting rewards,” prompting focus on past success,” “providing instructions,” “teaching to use prompts/cues,” and “prompting self-talk;” this cycle was the same for each of the 4 follow-up sessions. Figure 2 displays the flowchart of the follow-up sessions.

The effect of “MyPlan 2.0” will be tested by a randomized controlled trial. If the intervention is effective, it will be disseminated and implemented by the “Flemish Institute for Healthy Living,” which is the Flemish center of expertise regarding health promotion and illness prevention.

### Procedure

Participants were contacted by telephone and informed about the study. When participants decided to take part in the study, they received an email with a website link to the intervention and the documents to provide their informed consent. Participants were instructed to complete the intervention on their own. When researchers noted that participants forgot to log in at the scheduled time, they were reminded of doing so by a telephone call. After completing 5 intervention sessions, users’ website data were downloaded, and a date to perform a semistructured interview was scheduled. Before the start of the interview, participants completed questions about demographic characteristics (ie, age, gender, educational level, height, and weight). The interviews took place at the research department or via a telephone call. The interviews were audiorecorded with permission of participants.

The questions and content of the semistructured interview were based on the results of the previous qualitative research with the intervention “MyPlan 1.0” [30]. The 3 main topics that were addressed during the interview were as follows: design of the intervention (ie, general appreciation, user-friendliness, time efficiency, and layout); usefulness of the website (ie, opinion about the motivational value of the website, opinion about the informative value, feelings of awareness elicited by the website, personal relevance, and recommendations); and views about the benefits of being more physically active or less sedentary. During the discussion of each topic, researchers explicitly focused on how users had experienced each of the self-regulation techniques implemented in “MyPlan 2.0” (eg, “How did you experience the component in which you were asked to formulate personal barriers and solutions?”). The interview guide can be found in Multimedia Appendix 2. In the Results section, we will focus on perceptions’ regarding the website in general and the implementation of the behavioral change techniques. The average duration of an interview was 30 minutes, and participants received a reimbursement of €20.

### Data Analysis

The following information was derived from the users’ website data. First, we identified how many users selected sedentary behavior and physical activity as their target behavior and how many received the tailored feedback that they did not meet the respective health norm (ie, 30 minutes of, at least, moderate physical activity a day [39] or accumulating <8 hours of sitting time a day [40]). Second, time spent on the website and clicks on optional pages were calculated. Optional pages included the quiz, page about social support, and the extra tips describing techniques such as “prompting rewards,” “prompting a focus on past success,” “providing instructions,” “teaching to use prompts/cues,” and “prompting self-talk.” In addition, the average score on the quiz was calculated. Third, users’ action plans were checked by CVdM for achievability and instrumentality toward the chosen behavior [41,42]. Fourth, we calculated how many users were able to (partially) reach their goals and how many times the goals were adapted. Finally, barrier identification/problem solving was checked for achievability (ie, is it possible to execute the solution?) and instrumentality (ie, does the solution actually solve the identified problem?) by CVdM; for example, the solution “scheduling a moment in my diary” was coded as instrumental and achievable for the problem “I do not have enough time,” whereas this solution was considered achievable but not instrumental for the problem “I do not like to do it.”

Interviews were transcribed verbatim, and a directed content analysis was performed using NVivo Software (QSR International, Melbourne, Australia, Version 11, 2015) [43]. Content analysis is a way to comprise text into categories based on explicit coding rules [44-47]. In the directed content analysis, theory or prior research guides the coding. Directed content analysis is different from other strategies to analyze qualitative data in which codes most often emerge from the data [48]. Directed content analysis was considered best suited for our purpose because our coding scheme was based on previous research with “MyPlan 1.0” [30], and we were particularly interested in how participants precisely experienced the practical application of self-regulation techniques. Nevertheless, when a text fragment of the interview did not fit any of the predefined categories, a new category was created. Themes that did not contain enough data were not withheld. Coding was performed independently by two researchers (CVdM and LP). Furthermore, a weighted kappa was calculated, and it showed fair to good interrater agreement (weighted kappa=.67). Multimedia Appendix 3 shows an overview of the themes and subthemes. Multimedia Appendix 4 contains the completed COnsolidated criteria for REporting Qualitative research checklist [49].

## Results

### Demographic Characteristics

When contacted via telephone, 30 participants were willing to participate. However, 6 participants dropped out before the intervention period, and 4 participants did not respond to the researchers’ telephone calls. Recruitment was continued until 20 participants fully completed the 5 intervention sessions. Table 1 shows the demographic characteristics of 20 participants.

### Users’ Website Data

Table 2 shows the users’ website data according to both behaviors separately. Most users also visited the free-choice components, such as the quiz and additional pages regarding the social support. Only a small number of users indicated that they did not reach their goal or did not want to adapt their goal during the follow-up sessions. Almost all plans (38 of 40) were achievable and instrumental (eg, “On Monday and Wednesday I will perform my workout schedule at home”). The 2 exceptions were plans about sedentary behavior. In these plans, users indicated that they would perform a physical activity-related activity. During the first session, users had to select barriers and solutions from a list, which made all coping plans instrumental.

### Interviews

#### Website in General

In general, users stated that participating in the study and being involved in the intervention program raised awareness of their own behavior.

You are also made more aware, and that’s where it all starts.

Woman, >45 years, high educational level, normal weight

It was just the fact that I was more aware because I had to take a moment for it.

Woman, 18-45 years, high educational level, normal weight

Overall, the intervention website was perceived as user friendly and easy in use. Users highlighted the fact that it was clear and straightforward. In addition, the layout of the website was experienced as positive; it was simple and clear. Yet, some users would have liked a more colorful design.

**Table 1:** Demographic characteristics of participants.

Characteristics		Particpants (N=20)
**Gender, n (%)**		
	Men	10 (50)
	Women	10 (50)
**Age in years, mean (SD), range**		46.65 (16.65), 21-74
	18-45 y, n (%)	10 (50)
	>45 y, n (%)	10 (50)
**Educational level, n (%)**		
	Primary education	1 (5)
	Lower secondary education	1 (5)
	Higher secondary education	8 (40)
	College or university	10 (50)
**Body mass index (kg/m²), mean (SD), range**		25.42 (4.99), 18.47-37.81
	Not overweight, n (%)	11 (55)
	Overweight, n (%)	9 (45)

**Table 2:** Users’ website data according to the 2 target behaviors (sedentary behavior and physical activity).

Website Data		Total (N=20)	Sedentary behavior (n=8)	Physical activity (n=12)
Number of users receiving feedback of not reaching the health norm, n (%)		7 (35)	6 (75)	1 (8)
Time spent per session (min)		6.67	6.74	6.59
Number of users reading the extra tips, n (%)		16 (80)	6 (75)	10 (83)
Number of users reading more about social support, n (%)		14 (70)	4 (50)	10 (83)
Number of users taking the quiz, n (%)		20 (100)	8 (100)	12 (100)
Mean score on the quiz (out of 5)		4.4	4.71	4.08
Number of users willing to monitor their behavioral change, n (%)		15 (75)	4 (50)	11 (92)
Number of plans not achievable or instrumental, n (%)		2 (2)	2 (2)	0 (0)
**Indication of...during goal review, n (%)**				
	Total achievement	39 (49)	20 (63)	19 (40)
	Partial achievement	37 (46)	11 (34)	26 (54)
	Failure	4 (5)	1 (3)	3 (6)
**Choice to...their plan, n (%)**				
	Adapt	11 (14)	5 (16)	6 (12)
	Maintain	69 (86)	27 (84)	42 (88)
**Number of solutions not achievable or instrumental, n (%)**				
	Session 2	8 (40)	2 (25)	6 (50)
	Session 3	4 (20)	2 (25)	2 (17)
	Session 4	7 (35)	1 (13)	6 (50)
	Session 5	4 (20)	2 (25)	2 (17)

I thought it was a very good website. Very clear. I always knew what to do, where to click.

Woman, 18-45 years, high educational level, normal weight

It provided overview and was very clear. Nothing negative to mention. It was very easy, very simple. Yes, you could not do anything wrong I think.

Man, >45 years, high educational level, normal weight

I thought the layout was simple, but that didn’t bother me. I think it contributed to the clarity.

Man, 18-45 years, high educational level, normal weight

In line with that, users also stated that they would have liked more interaction on the website and more new content per session. For some users, the website was too repetitive and could have been more appealing. Yet, most of the participants were positive about the website and the initiative in general.

I think, if people will visit the website regularly, they will want to see something new every time though.

Woman, >45 years, low educational level, overweight

It is useful that you try to let people be physically active. You can think about it yourself, everything comes from you. There is no one telling you: ‘You have to do this if that happens’. You give yourself feedback.

Woman, 18-45 years, low educational level, overweight

Almost all participants experienced the intervention as personally relevant and appropriate. However, the website seemed less fitting for persons who considered themselves as being physically active or for individuals with a lack of motivation.

It is developed generation-independently, from 7 until 77 in a manner of speaking.

Man, >45 years, low educational level, overweight

Normally, I am already physically active. In that way, the added value for me was minimal. Maybe the intervention is too restricted because it is assumed that people experience difficulties in being physically active.

Man, >45 years, high educational level, normal weight

Most users appreciated the time efficiency of the website. Some users would have liked a little more content and for other users, content could have been shown in even less internet pages.

That (cf. the length) was very reasonable. Certainly not too long. However, not too short either. I had expected a lot more questions and other things.

Man, >45 years, high educational level, overweight

In addition, the intervention was perceived as motivating and stimulating for behavioral change by most users. However, some users experienced problems putting their intention into action. Other users were not motivated enough to change their behavior.

It is stimulating to initiate behavior.

Man, >45 years, low educational level, overweight

The website totally helped me, because I wasn’t exercising anymore at all and now I am exercising again. So it did work.

Man, 18-45 years, low educational level, overweight

It is a very good initiative, but it is still difficult to translate it into action and actually move more or sit less. It seems evident, but it is not.

Man, 18-45 years, high educational level, normal weight

#### Goal Setting

Users often mentioned that the difference between physical activity and sedentary behavior was not clear for them, which made the intervention more complex.

For me there was little difference. If you sit less, then you automatically move more, and if you move more, then you sit less. So I didn’t think it was clear.

Woman, >45 years, high educational level, normal weight

#### Providing Information on the Consequences of the Behavior

All participants stated that being more physically active or less sedentary has benefits for both physical and mental health. Some participants believed in the benefits but indicated that they had not experienced the benefits because of the intervention.

I think it has an influence. I really believe it has, but I have not experienced it.

Woman, 18-45 years, high educational level, normal weight

Accordingly, most users indicated that they did not learn new things through the intervention. They already knew the consequences of their behavior. They only had to be reminded to do something about it.

Learned new things? No. But it gave new insights, you take a moment to think about it.

Man, >45 years, high educational level, normal weight

#### Providing Feedback on Performance

The tailored feedback was highly appreciated by users. They recommended such feedback as the first step toward behavioral change. According to users, the feedback was personally tailored and made them aware that they had to change their behavior. Some users found that the feedback stimulated them actually to alter their behavior. Other users did not remember the feedback from the first session.

It was good to know where you are because you really don’t have a clue.

Woman, 18-45 years, high educational level, overweight

I thought it (cf. the feedback) was good. That way, you know where you are and where you can improve. And it is different for every person. So, it is more personal.

Woman, 18-45 years, high educational level, normal weight

#### Action Planning

Action planning was experienced as highly motivating. Users appreciated the fact that they could plan their personal goals in a structured way by questions. Many users indicated that they actually performed their goal as planned.

I think it is important to plan this. Because everyone is busy and otherwise there is always something else coming up. If you don’t make it a goal or plan in your week, it will not occur or it will fade with time.

Woman, >45 years, high educational level, normal weight

So putting my mobile phone further away (cf. in order to decrease sedentary time) is something that I do now.

Man, 18-45 years, low educational level, normal weight

Some users reported problems with action planning. They thought it was difficult to plan behavioral change a week in advance, especially when they had changing work hours. Furthermore, they preferred planning using a calendar rather than by questions. Other users found it difficult to plan behavioral change because they lacked the knowledge and inspiration about what to do. They wanted ready-to-use activity programs.

If you know what you want to do, but you do not put the words into action, then you fill this in. However, if someone knows he wants to be more physically active, but hedoes not know how exactly, then I think he will ask himself: “What should I do now?”

Man, 18-45 years, high educational level, normal weight

At the beginning I found it difficult to set up goals for myself.

Woman, 18-45 years, high educational level, normal weight

#### Barrier Identification/Problem Solving

Most users found it a good idea to think about barriers in advance and try to find solutions. However, many indicated that it was difficult to anticipate what could go wrong and how to overcome problems. Users expected the website to provide more guidance for this component.

What I really appreciated, is the fact that you were obliged to write down at least one barrier and how to cope with it. I had to take a bit of time to think about it, but in the end I always found one. The barrier component is the most powerful of the intervention.

Man, 18-45 years, high educational level, normal weight

Sometimes it was difficult. Because experiencing barriers is not difficult, but finding solutions is not always easy. Most of the time, the same barriers arose.

Woman, 18-45 years, high educational level, normal weight

Barrier identification really was something else (cf. in comparison to action planning). You have to be able to think immediately about what hinders you. That was more difficult. And maybe there could have been more guidance from the website.

Woman, >45 years, low educational level, overweight

#### Prompting Self-Monitoring of Behavior

Many users misunderstood the purpose of self-monitoring and wrote down their plan in advance to remind them about it, but did not keep track of whether they executed the planned behaviors or not.

I always wrote it down in my diary, in color. That is definitely useful, otherwise you forget about it.

Man, 18-45 years, high educational level, normal weight

I had expected that I would be assisted to monitor my goals myself, to see how my sitting time changes. But I was not asked to write down my sitting time.

Man, 18-45 years, high educational level, normal weight

#### Plan Social Support

There were a few users who commented on the social support component. Some users found it very useful to involve others, whereas other users preferred to keep their behavioral change more private.

I also appreciated the more practical tips such as inviting neighbors or not exercising alone. I found it nice to read and I often took it into account.

Women, 18-45 years, high educational level, normal weight

I did not really like the social parts. I prefer to do this on my own.

Woman, >45 years, low educational level, normal weight

#### Prompt Review of Behavioral Goals

The largest group of users found it useful to review their goals. Many users indicated that having to log in again was the most motivating part of the intervention.

The good thing was that it repeated itself every week. Another program ends after one session and then you have the tendency to put it aside. Since you had to log back in for five weeks, you wanted to do what they asked because they would ask if you did it.

Woman, >45 years, low educational level, overweight

#### Tips

Most users expressed their interest in the extra tips and found them very useful. The tips were experienced as feasible and inspiring. Especially, the tip regarding “using prompts or cues” was often implemented. Some users indicated that more new tips during the sessions were needed. Reading success stories of other possible users was also perceived as of added value to the website, although some stated that the stories were too predictable.

The tips were very interesting because they were practically feasible. It were simple tips that were achievable.”

Woman, >45 years, high educational level, normal weight

It is always motivating to see (cf. read) how someone else does it, then you also want to motivate yourself to do it.

Woman, >45 years, low educational level, overweight

The most helping was the note on the fridge. It made you aware to not forget about your plans that day.

Man, >45 years, low educational level, normal weight

## Discussion

Web-based interventions are increasingly used to alter health behaviors [10] and have shown to be more effective when grounded in a solid behavioral change theory [11]. However, the high levels of attrition highlight the importance to also target user engagement [36]. User engagement has been defined and measured in many ways [50]. According to Perski et al., engagement with a Web-based intervention is influenced by context (eg, the demographic characteristics of the population) and intervention (eg, the complexity of the intervention) variables [51]. This study focuses on the latter by investigating how users experienced a self-regulation-based eHealth intervention targeting physical activity and sedentary behavior. Users’ website data were analyzed, and 20 semistructured interviews were performed.

Besides investigating users’ opinions about self-regulation techniques, we also explored how they perceived the intervention in general. In comparison with the users of “MyPlan 1.0” [30], those of the 2.0-version appreciated the time efficiency and user-friendliness of the program; this is encouraging because it proves that an iterative approach in which users are consulted during the development of the intervention pays off [26]. Intervention developers should keep an eye on the user-friendliness of their intervention. We found that a simple but agreeable layout enhanced user-friendliness. Likewise, previous research has indicated that professional design and simple navigation can increase engagement [52]. Some users suggested that the development of a similar mobile app may further increase user-friendliness and interactivity; this suggestion is in line with research showing that the use of mobile apps might increase the adherence [53]. Most users perceived the sessions’ duration of approximately 5 minutes as a perfectly reasonable length. Intervention developers are already aware that eHealth interventions should be kept short and to the point [32,52] but reducing length while still implementing different self-regulation techniques has not been an easy endeavor.

This study revealed that most users were well aware of the benefits of increasing physical activity or reducing sedentary behavior; this was reported in the interviews. Users often mentioned that the intervention did not substantially increase their knowledge about the beneficial effect of a more active lifestyle, and this finding was corroborated by the high scores on the quiz, which aimed to provide information engagingly. Notwithstanding, users were interested in information and all completed the optional quiz. The findings indicate that further tailoring and offering more advanced information is recommended in this target population. In addition, previous research highlights the importance of providing new information tailored to the users’ needs [32]; for example, Short et al. stated that offering personalized information could increase men’s engagement in a Web-based intervention targeting physical activity and nutrition [54]. Of further interest, reading information and receiving personal feedback on the questionnaires seemed to function as a prompt to behavioral change; it reminded users about the importance of adopting a more active way of living.

Of particular interest to this study were the experiences and opinions of users about the self-regulatory strategies to bridge the intention-behavior gap. Key to our eHealth intervention were action planning and problem solving. Action planning consisted of formulating specific actions and planning about when and how they will conduct these behaviors. Action planning seemed to be feasible. Few users stated unachievable plans and many were able to reach their goals, at least, partially. However, thinking in advance about actions was experienced as difficult and effortful by users. Some stated that it was difficult to come up with specific actions or plan these actions a week in advance, and this is a good remark. An improvement may be to allow users to create and evaluate specific plans on a daily basis. Implementing such microcycles might offer users more guidance in creating instrumental and achievable plans on a daily basis.

The implementation of the technique “barrier identification/problem solving” was less feasible. Many users struggled with identifying barriers and finding solutions in advance, especially in the follow-up sessions in which they had to answer an open-ended question; this was communicated in the interviews and further corroborated by the analysis of the provided barriers and solutions at the website. Our results seem to be at odds with those of other studies. Sniehotta et al. [55] successfully implemented this technique in their intervention to increase physical activity in cardiac rehabilitation patients; their implementation of the technique was very similar to ours—participants were asked which barriers could interfere with their plans and how they could successfully cope with these barriers. However, an important difference with our study is that trained consultants helped users with problem solving in face-to-face contact. Indeed, self-regulation techniques have mostly been used in face-to-face settings [13]. It may well be that counselors are better able to adapt to the implementation of these techniques to the context and needs of an individual. To date, Web-based interventions do not easily offer such an opportunity, and this is an issue worth further consideration and follow-up. Effective techniques may become useless (or even counterproductive) when their implementation is or remains suboptimal. Based on these findings, we recommend offering sufficient guidance when implementing the “barrier identification/problem solving” technique; for example, a button saying “need help?” was added in “MyPlan 2.0” When clicking on this button, users are shown an extensive list of potential barriers and solutions, which can guide them to answer the open-ended question.

In the interviews, some participants mentioned that the intervention may be of lesser use for individuals who are not ready for change yet, and this view is in line with various theoretical models of behavioral change, such as the Stages of Change Theory [56] and the Health Action Process Approach [57]. According to these models, individuals who are not ready to change will not engage in action programs. Indeed, studies investigating engagement according to user characteristics show that users’ level of motivation is an important factor for the eHealth uptake [58]. Interventions targeting these individuals might then better include techniques such as motivational interviewing [59], focusing on raising awareness, and eliciting change talk. Such motivational techniques were largely absent in our intervention. We reasoned that eHealth interventions were relatively inadequate for participants with low motivation to change behavior in the short term. Perhaps, more intensive interventions, including face-to-face contact, may be more suited for these individuals [60].

In addition, users indicated that the intervention might be of lesser use for individuals who already have a habit of being active. Inadvertently, many of our participants already had an active way of living. Their personal feedback on the questionnaire stated that they reached the health norm. We had opted not to exclude participants who reached the health norms. First, research has demonstrated that individuals often overestimate their activity levels when self-report measures of physical activity are used [61]. We reasoned that participants may become more accurate of their estimations of physical activity by engaging in the intervention. Second, we reasoned that the eHealth intervention might also help in maintaining the behavior of those who are already habitually active, but this might not be the case. These individuals may experience the action and coping plan as needlessly effortful, frustrating, and cumbersome. Consequently, informing users explicitly about the target group of the intervention might be worth considering.

One of the strengths of this study was the diversity of the sample with an equal distribution of gender, age, educational level, and body mass index. Furthermore, having both users’ website data, as well as interview data, strengthened our conclusions. Finally, the perspective of users on the specific implementation of self-regulation techniques has not been often investigated. The most important limitation of this study was the fact that we did not investigate the participants’ actual levels of physical activity and sedentary behavior using validated methods. Consequently, we do not know whether our sample was more active than the general population. In addition, we were unable to assess the experiences of 4 users who quit the intervention. It may well be that their experience with the intervention was less positive. Furthermore, participants who were acquaintances of researchers might have had a more positive perception of the eHealth intervention. However, to limit this impact, these participants were always interviewed by a trained researcher they did not know.

In conclusion, this study reveals that behavioral change theories may be necessary but not sufficient to guarantee the efficacy in designing interventions. Equally important is the involvement of end users [25-27] because they can inform intervention developers on how self-regulation techniques should (or should not) be integrated. To ameliorate users’ engagement with a Web-based intervention, we have the following recommendations: create short (5-6 minutes) interventions with a straightforward layout; provide novel and tailored information regarding the benefits of the health behavior; make users create specific action plans and review these plans in the follow-up sessions; and provide guidance and practical examples when adding a problem solving module.

## Appendix

Multimedia Appendix 1 Implementation of the behavior change techniques in the website.

Multimedia Appendix 2 The Interview Guide.

Multimedia Appendix 3 Overview of the themes and subthemes.

Multimedia Appendix 4 Completed COREQ checklist.

## Abbreviations

IPAQ: International Physical Activity Questionnaire
