# Supplementary material for: How Users Experience and Use an eHealth Intervention Based on Self-Regulation: Mixed-Methods Study
Source: J Med Internet Res. 2018 Oct 1;20(10):e10412. doi: 10.2196/10412 (PMC6231831; doi:10.2196/10412)
Supplement: Multimedia Appendix 1 [file jmir_v20i10e10412_app1.pdf]

Implementation of the self-regulation techniques in MyPlan 2.0

Providing information on the consequences of behaviour

The website offers the option to take a quiz regarding the chosen health behaviour (i.e. increasing physical activity or decreasing sedentary behaviour). The quiz consists of five statements about the positive effects of the chosen behaviour. Each solution is accompanied by a short and easy text describing the scientific research that has been carried out to answer the question.

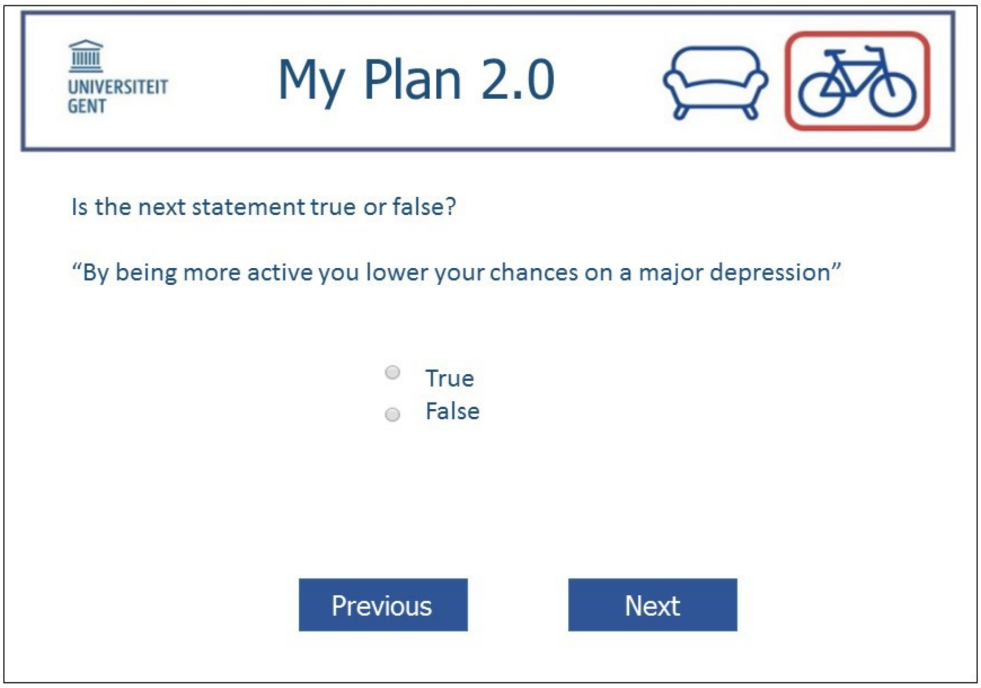

Figure 1. Example of a quiz question

Providing feedback on performance

Participants complete a short questionnaire regarding the selected health behaviour (i.e. a shortened version of the International Physical Activity Questionnaire (IPAQ) or a last 7-days sedentary behaviour questionnaire) and receive tailored feedback.

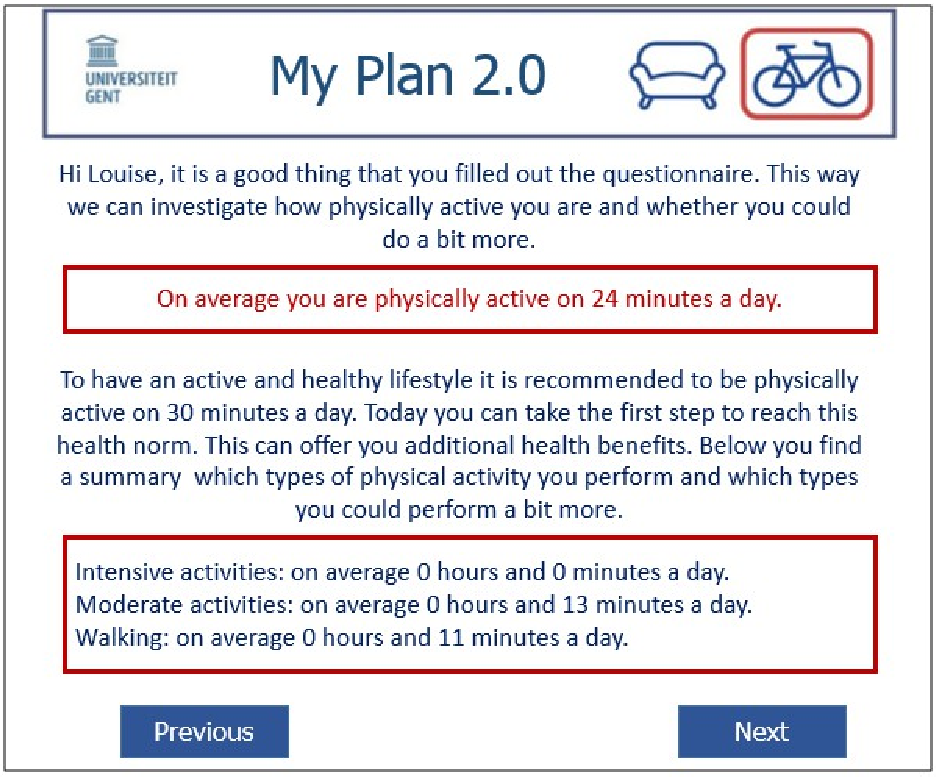

Figure 2. Example of the tailored feedback

Action planning

During this component, users specify their actions in terms of what, where and when by answering open and multiple choice questions. Several tips are provided to make the action plan feasible (e.g. “Choose for one goal instead of multiple goals, this increases the chance of goal attainment”).

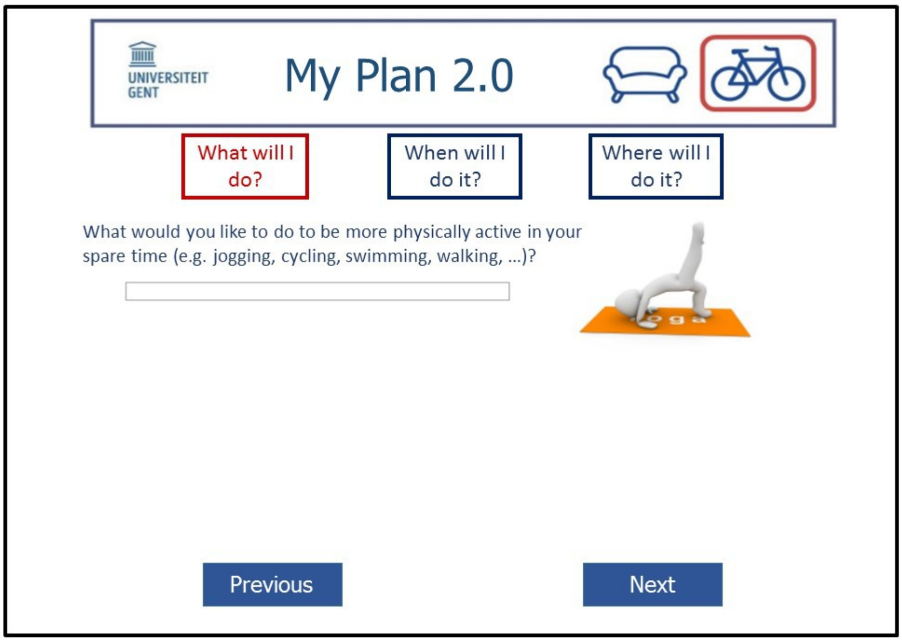

Figure 3: Example of a question in the action planning component

Barrier identification / Problem solving

'Barrier identification/problem-solving' is implemented by asking users which barriers they can perceive and which solutions are possible to overcome these barriers.

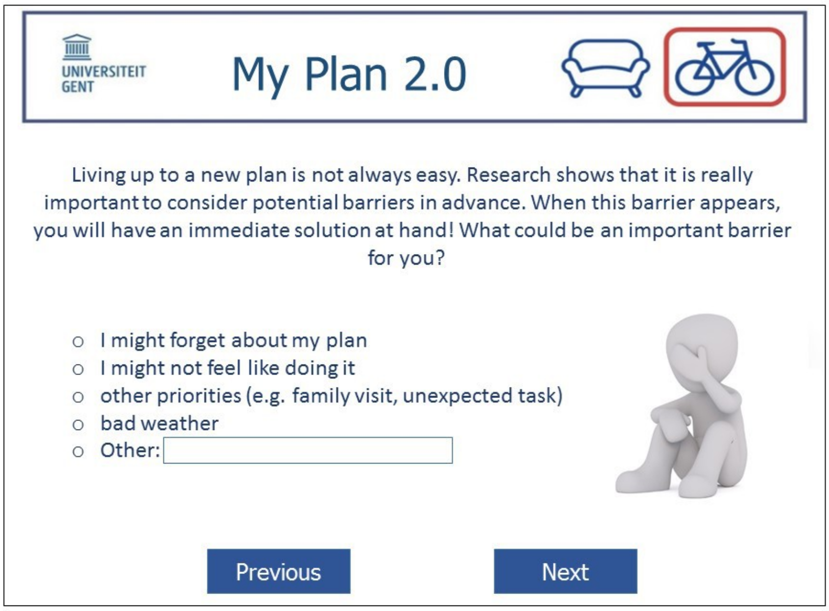

Figure 4. Barrier identification / problem solving

Prompting self-monitoring

Self-monitoring is prompted by letting users choose from a list how they will monitor their own behaviour (e.g. via their calendar, in a notebook, ...).

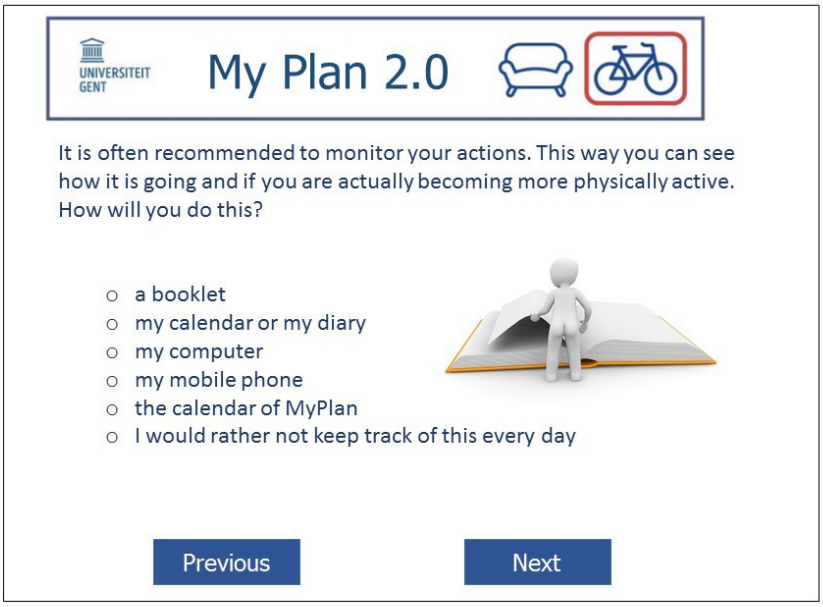

Figure 5: Prompting self-monitoring

Planning social support

At the end of the first session ‘planning social support’ is introduced: users read about how to elicit social support, how to talk about behaviour change to significant others, and how to find opportunities to engage in behaviour change together with other people.

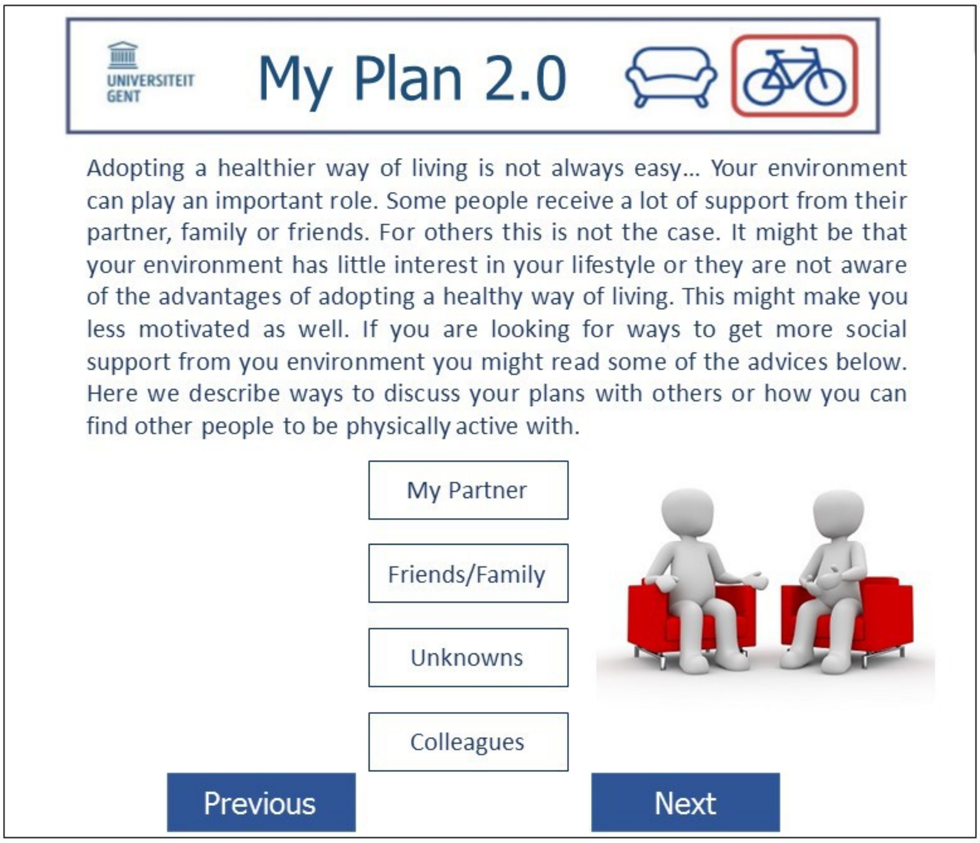

Figure 6: Planning social support
