# Supplementary material for: How Users Experience and Use an eHealth Intervention Based on Self-Regulation: Mixed-Methods Study
Source: J Med Internet Res. 2018 Oct 1;20(10):e10412. doi: 10.2196/10412 (PMC6231831; doi:10.2196/10412)
Supplement: Multimedia Appendix 2 [file jmir_v20i10e10412_app2.pdf]

Interview Guide

- Which module did you select, less sitting or being more physically active?

<u>Design of the website</u>

Perception of the website

- In general, how do you feel about the website?
- How did you experience using the website?
- To what extent, did you perceive the website as engaging?
- To what extent, did you think using the website was a fun thing to do?
- Could you easily keep your attention to the website?
- Which parts of the website did you like the most?
- Which parts of the website did you like the least?
    - Did you like the questionnaire and its accompanying feedback?
    - Did you like creating an action plan?
    - Did you like searching for barriers and solutions?
    - Did you like reading the tips (e.g. regarding social support)?
    - Did you like monitoring your goal?

User-friendliness

- In general, was the website easy or difficult to use?
- Which parts of the website did you experience as easy?
- Which parts of the website did you experience as difficult?
- How do you think about the user-friendliness of the website?
    - Could you easily find what you needed?
    - Where there moments that you were stuck?
    - Could you easily read all the text?
- Was the questionnaire and it accompanying feedback easy/difficult?
- Did you perceive creating your own action plan as easy/difficult?
- Did you perceive searching for barriers and solutions as easy/difficult?
- Were the tips easy/difficult?
- Did you perceive monitoring your goal as easy/difficult?

Time efficiency

- What do you think about the time you needed to complete the sessions?

Lay-out

- What do you think about the lay-out and the design of the website?

<u>Usefulness of the website</u>

Recommendations

- Do you suggest any adaptations to the website?

- Are there things you would recommend us to change regarding the website?

Stimulating value of the website

- To what extent did the website help you to change your behaviour?
- Which parts of the website helped you the most?
- Which parts of the website helped you the least?
  - Was it helpful to create an action plan?
  - Was it helpful to search for barriers and solutions?
  - Was it helpful to read the tips (e.g. regarding social support)?
  - Wat is helpful to monitor your goal?

Awareness

- To what extent did the website make you more aware?

Personal relevance

- Did you perceive the website as personal relevant?
- Did you perceive the questionnaire and its accompanying feedback as personal relevant?

Informing value of the website

- To what extent did you learn new things by using the website?
- To what extent did you learn new things regarding behaviour change?
- To what extent did you learn new things regarding being less sedentary or more physically active?

Knowledge

- Do you think sitting less/being more physically active has an influence on how feel physically/mentally?
- Do you think sitting less/being more physically active has an influence on your health?
