# Supplementary material for: How Users Experience and Use an eHealth Intervention Based on Self-Regulation: Mixed-Methods Study
Source: J Med Internet Res. 2018 Oct 1;20(10):e10412. doi: 10.2196/10412 (PMC6231831; doi:10.2196/10412)
Supplement: Multimedia Appendix 3 [file jmir_v20i10e10412_app3.pdf]

Overview of the themes and subthemes

Self-regulation technique		Number of participants mentioning it positively	Number of participants mentioning it negatively
**Website in general**			
	**Awareness**	19	5
	**User-friendliness**	20	16
	**Lay-out**	17	12
	**General perception**	19	16
	**Personal relevance**	17	11
	**Time-efficiency**	20	11
	**Motivational value**	19	14
**Goal setting**		0	6
**Providing information on the consequences of the behaviour**			
	**Informing value**	11	16
	**Knowledge**	20	7
**Providing feedback on performance**			
	**Awareness**	9	1
	**Personal relevance**	11	1
	**Motivational value**	9	7
**Action planning**			
	**Motivational value**	19	7
	**User-friendliness**	17	10
**Barrier identification/problem-solving**			
	**Motivational value**	14	11
	**User-friendliness**	9	13
**Prompting self-monitoring of behaviour**			
	**User-friendliness**	7	9
	**Motivational value**	15	15
**Plan social support**			
	**Motivational value**	4	3
**Prompt review of behavioural goals**			
	**Motivational value**	8	0
**Tips: prompting rewards, prompting focus on past success, providing instructions, teaching to use prompts/cues and prompting self-talk**			

	**User-friendliness**	12	9
	**Motivational value**	18	13
