# Supplementary material for: How Users Experience and Use an eHealth Intervention Based on Self-Regulation: Mixed-Methods Study
Source: J Med Internet Res. 2018 Oct 1;20(10):e10412. doi: 10.2196/10412 (PMC6231831; doi:10.2196/10412)
Supplement: Multimedia Appendix 4 [file jmir_v20i10e10412_app4.pdf]

Consolidated criteria for reporting qualitative research (COREQ): a 32-item checklist for interviews and focus groups

Domain 1: Research team and reflexivity

Personal characteristics

1. Interviewer/facilitator. Which author/s conducted the interview or focus group?

LP, CVDM and two Master students (trained by LP and CVDM) conducted the interviews.

2. Credentials. What were the researcher's credentials? E.g. PhD, MD

LP: PhD candidate in Health Sciences and Psychology
CVDM: PhD candidate in Psychology and Health Sciences
IDB: PhD in Psychology
MV: PhD in Physical Education
GC: PhD in Psychology

3. Occupation. What was their occupation at the time of the study?

LP and CVDM are PhD students performing research; MV is a postdoctoral researcher in health promotion, IDB is full professor in health promotion. GC is full professor in Health Psychology.

4. Gender. Was the researcher male or female?

LP, CVDM, IDB, and MV are female researchers, whereas GC is a male researcher.

5. Experience and training. What experience or training did the researcher have?

LP has a Master's degree in Experimental and Theoretical Psychology.

CVDM has a Master's degree in Clinical Psychology.
IDB has a Master's degree in Clinical Psychology and a PhD in Health Psychology
MV has a Master's degree and PhD in Physical Education and Movement Sciences
GC has a Master's degree in Clinical psychology and a PhD in Psychology.

Relationship with participants

6. Relationship established. Was a relationship established prior to study commencement?

No relationship with most of the participants was established before the commencement of the study. However, some of the participants were acquaintances of the interviewers.

7. Participant knowledge of the interviewer. What did the participants know about the researcher? e.g. personal goals, reasons for doing the research

The participants knew that the interviewers created 'MyPlan 1.0' and conducted the study in order to ameliorate the programme.

8. Interviewer characteristics. What characteristics were reported about the interviewer/facilitator? e.g. Bias, assumptions, reasons and interests in the research topic

Specific characteristics of the researchers (e.g. training, profession) can always have an influence on data collection and analysis. Nevertheless, we created strict protocols to carry-out the interviews and to analyse the data to minimize bias.

Domain 2: study design

Theoretical framework

9. Methodological orientation and Theory. What methodological orientation was stated to underpin the study? e.g. grounded theory, discourse analysis, ethnography, phenomenology, content analysis

A directed content analysis was conducted. This type of analysis was considered best suited for our purpose because our coding scheme was based upon previous research with 'MyPlan 1.0'

Participant selection

10. Sampling. How were participants selected? e.g. purposive, convenience, consecutive, snowball

The sample from the general population was recruited via an available database, consisting of individuals who had expressed their interest to participate in studies of the Ghent Health Psychology Research Group via a website

(http://www.healthpsychology.ugent.be/vrijwilligers), and via the snowball sampling technique.

11. Method of approach. How were participants approached? e.g. face-to-face, telephone, mail, email

Participants were recruited in different ways: face-to-face, telephone and email.

12. Sample size. How many participants were in the study?

When phoned, thirty participants were willing to participate. However, six participants dropped out before the intervention period, and four participants did not respond to the researchers telephone calls. Recruitment was continued until twenty participants fully completed the five intervention sessions.

13. Non-participation. How many people refused to participate or dropped out? Reasons?

Six participants dropped out before the intervention period, and four participants did not respond to the researchers telephone calls.

Setting

14. Setting of data collection. Where was the data collected? e.g. home, clinic, workplace

The interviews took place at the research department or via a telephone call. The interviews were audio-recorded with permission of the participants.

15. Presence of non-participants. Was anyone else present besides the participants and researchers?

We asked participants to conduct the interview in a room where they would not be disturbed. However, in some cases we could not prevent that a family member occasionally disturbed the interview.

16. Description of sample. What are the important characteristics of the sample? e.g. demographic data

The demographic information of the sample is provided in table 1 of the manuscript.

Data collection

17. Interview guide. Were questions, prompts, guides provided by the authors? Was it pilot tested?

All questions are provided in additional file 2.

18. Repeat interviews. Were repeat interviews carried out? If yes, how many?

There were no repeat interviews carried out.

19. Audio/visual recording. Did the research use audio or visual recording to collect the data?

All verbalizations were voice-recorded.

20. Field notes. Were field notes made during and/or after the interview or focus group?

No.

21. Duration. What was the duration of the interviews or focus group?

The average duration of an interview was 30 minutes.

22. Data saturation. Was data saturation discussed?

When a text fragment of the interview did not fit any of the predefined categories, a new category was created. Themes that did not contain enough data were not withheld. Coding was done independently by two researchers (CV and LP). A weighted kappa was calculated and showed fair to good inter-rater agreement (weighted kappa: 0.67).

23. Transcripts returned. Were transcripts returned to participants for comment and/or correction?

No.

Domain 3: analysis and findings

Data analysis

24. Number of data coders. How many data coders coded the data?

Two data coders (LP and CVDM) coded the data.

25. Description of the coding tree. Did authors provide a description of the coding tree?

Yes. This is provided in additional file 3.

26. Derivation of themes. Were themes identified in advance or derived from the data?

The themes were identified in advance. This was based on previous research with “MyPlan 1.0”.

27. Software. What software, if applicable, was used to manage the data?

The qualitative data analysis software nVivo 11 (QSR International Pty. Ltd. Version 11, 2015) was used to manage the data.

28. Participant checking. Did participants provide feedback on the findings?

No.

Reporting

29. Quotations presented. Were participant quotations presented to illustrate the themes / findings? Was each quotation identified? e.g. participant number

Yes.

30. Data and findings consistent. Was there consistency between the data presented and the findings?

Yes.

31. Clarity of major themes. Were major themes clearly presented in the findings?

Yes. Additional file 3 gives an overview of the amount of positive and negative references per theme.

32. Clarity of minor themes. Is there a description of diverse cases or discussion of minor themes?

Yes.
